# Investigation of Bioinspired Nacreous Structure on Strength and Toughness

**DOI:** 10.3390/biomimetics7030120

**Published:** 2022-08-27

**Authors:** Biao Tang, Shichao Niu, Jiayi Yang, Chun Shao, Ming Wang, Jing Ni, Xuefeng Zhang, Xiao Yang

**Affiliations:** 1 School of Mechanical Engineering, Hangzhou Dianzi University, Hangzhou 310018, China; 2 Key Laboratory of Bionic Engineering, Ministry of Education, Jilin University, Changchun 130022, China; 3 The School of Technology, Beijing Forestry University, Beijing 100083, China; 4 College of Materials and Environmental Engineering, Hangzhou Dianzi University, Hangzhou 310018, China

**Keywords:** extended finite element, nacre, structural parameters, strength and toughness

## Abstract

The toughening mechanism of the nacre was widely investigated in recent decades, which presents a great prospect for designing high performance composite materials and engineering structures with bioinspired structures. To further elucidate which structural parameters and which kinds of morphology of the nacre-inspired structure are the best for improving tensile strength without sacrificing too much toughness is extremely significant for composite materials and engineering structures. The “brick-and-mortar” structure is a classical nacre-inspired bionic structure. Three characteristic structural parameters, including the aspect ratio ρ of the brick length and width, the thickness ratio β between the thickness of brick and mortar, and the spacing ratio τ between the width of brick and mortar, were used as variables to study their effect on tensile strength and toughness. It was found that ρ was the most prominent factor in determining the strength and toughness, and τ could improve the strength and toughness almost simultaneously. Racked and wedged morphology of the structural unit were established based on the structural parameters of the regular staggered unit, and were used to compare tensile behavior. It was found that the model with the wedged unit possessed the highest strength and toughness, and could absorb more strain energy during fracture crack growing. The crack propagation path further illustrated that the crack resisting ability of the wedged unit was the best. Our simulation results presented the connection between three characteristic structural parameters with the strength and toughness, and proved that the wedged staggered unit was the best in improving the strength and toughness.

## 1. Introduction

Higher strength and higher toughness still are two basic requirements for most engineering materials and structures, but they are mutually exclusive and can be barely achieved by single engineering material or structure [1,2]. It is a great challenge for researchers to solve this conflict between toughness and strength. Through millions of years’ evolution, some biological materials found in nature, such as bone, horn, teeth, and mollusk shell, show remarkable mechanical performance as a result of the sophisticated structures organized over several scales, which almost provide the perfect solutions to this conflict [3]. The nacreous layer of abalone or oyster is one of these attractive biological materials which is assembled by brittle inorganic components and soft organic materials, but exhibits almost twice the strength and is 1000-fold tougher than its main constituent, aragonite. Thus, it is meaningful to reveal the toughing mechanism of nacreous layer base on which the artificial composites are designed and are widely applied in various fields [4,5].

Numerous researchers made much work related to the materials compositions, microstructure, and mechanical performance of nacreous layers. It was found that the nacre was composed of 95 wt.% aragonite (a crystallographic form of CaCO_3_) and 5 wt.% organic materials (proteins and polysaccharides) [6,7]. The nacre has a staggered topological structure in which the hard phase CaCO_3_ is embedded into the soft phase organic materials, which is called “brick-and-mortar” (B-M) structure [8,9]. To be more specific, many little pores were found randomly distributed within the organic layer between each aragonite platelet. Through the porous organic layer, some mineral bridges connected adjacent aragonite platelets [10,11,12]. Moreover, aragonite platelets have a corrugated surface which was demonstrated, that played an important role in strain hardening when large deformation of the nacre emerged or while platelets were pulled out [13,14,15,16]. Some bioinspired mechanical models and crack resistant mechanisms were established and were revealed based on mechanical tests of nacreous layers. A tension-shear chain model of biological nanostructures inspired by nacre was used to reveal that the strength of biomaterials hinges upon optimizing the tensile strength of the mineral crystals. The optimized tensile strength of mineral crystals allowed a large amount of fracture energy to be dissipated in protein via shear deformation and consequently enhanced the fracture toughness of biocomposites [17]. A shear-lag model was also used to investigate the effect of structural parameters and components properties on the mechanical performance, such as strength, toughness, and stiffness. Crack bridging, deflection, and branching were found along the interface between the hard phase and soft phase [18,19].

Although these studies and models explained the fracture mechanism of nacreous layers to some extent, there were no definite models that indicated which kind morphology of the staggered unit and which group of structural parameters were the best in improving the tensile strength without sacrificing toughness. In this paper, the extended finite element method (XFEM) was used to explore the effect of structural parameters on strength and toughness, and revealed the toughening mechanism of different morphology of the basic staggered unit.

## 2. XFEM and Models

### 2.1. XFEM

XFEM based on the traction-separation law was used to simulate the crack propagation and failure process of the nacre, and several optimization indexes such as strength and toughness were calculated. XFEM was a recent numerical method based on the idea of elements’ decomposition in the conventional finite element displacement model, which added a jump function and a crack tip progressive displacement field to reflect the displacement discontinuity [20]. XFEM was first proposed and developed by Belytschko et al. [21] and Moës et al. [22] to solve the problems of discontinuity and singularity in the material field. Modeling discontinuities as an enriched feature using the XFEM, the crack-independent enrichment function of the mesh was represented as an approximation of the displacement vector function *u*, denoted as Equation (1):

(1) $$u\left(x\right)=\overset{N}{\underset{i=1}{\sum }}N_{i}\left(x\right)\left[u_{i}\right]+H\left(x\right)a_{i}+\overset{4}{\underset{\upsilon =1}{\sum }}F_{\upsilon }\left(x\right)b_{i}^{\upsilon }$$

In the above enrichment function, *N_i_ (x)* was the node shape function of elements in the B-M FEM model, *u_i_* was the displacement vector of the node in the element, *H(x)* was the discontinuous jump function inserted to form the crack path in the simulation structure, $F_{\upsilon }\left(x\right)$ was the asymptotic functions controlling the crack tip in the model, *a_i_* and $\;b_{i}^{\upsilon }$ were the vector of node enrichment freedom degrees. The above enrichment functions included tip asymptotic functions and discontinuity functions. Since the use of asymptotic crack tip functions was not limited to modeling cracks in isotropic elastic materials, it was particularly applicable to cracks in bi-material interfaces. The meshes used by XFEM were independent of the geometry or physical interface inside the structure [23], thus overcoming the difficulties brought by high-density mesh generation in high stress and deformation concentration areas such as the crack tip, and there was no need to regenerate the meshes when simulating crack propagation. The level set method was used to determine the actual position of the crack and track the growth of the crack, optimized the shape function of the element in the crack affected area [24]. The “elements decomposition” feature [25] makes the form of the stiffness matrix of the extended finite element the same as that of the conventional finite element.

**Figure 1:**
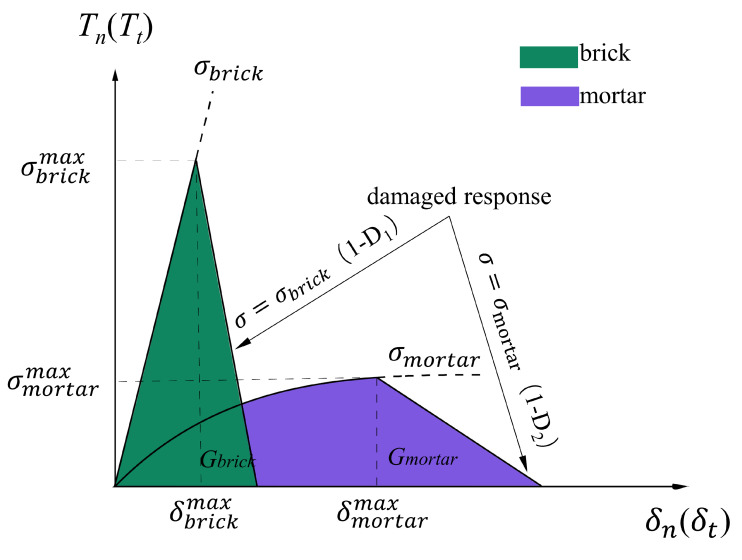
Damage evolution used in TSL.

Material failure followed the traction-separation law (TSL) specified in the cohesive zone model, where damage began when the material reached the defined initial damage stress or strain. An illustration of the traction-separation law is shown in Figure 1.

The damage initiation criterion was represented by Equations (2) and (3):

(2) f1=(σbrick)σbrickmax

(3) f2=(σmortar)σmortarmax

where $\sigma_{brick}$ and $\delta_{brick}^{max}$ were the maximum principal stress and maximum principal strain at the onset of damage in brick materials, $\sigma_{mortar}$ and $\delta_{mortar}^{max}$ were the maximum principal stress and maximum principal strain at the onset of damage in mortar materials. If *f* = 1 then a crack initiated, the stress in the element began to degenerate.

The damage evolution of the material followed the Beneggagh–Kenane (BK) law as shown in Equation (4):

(4) $$G_{C}=G_{IC}+\left(G_{IIC}-G_{IC}\right){\left(\frac{G_{II}+G_{III}}{G_{I}+G_{II}+G_{III}}\right)}^{\varphi }$$

where $G_{I}$, $G_{II}$, $G_{III}\;$ represented normal and shear fracture energy;φ was a cohesive property parameter. With regard to B-M, the critical fracture energy used in the XFEM model were specified to be $G_{IC}$ = $G_{IIC}$ = $G_{IIIC}$. When the fracture energy $G_{\mathrm{brick}}$ and $G_{mortar}$ of the B-M damage evolution were defined, the stresses in the material elements began to degrade linearly with the principal strain, the damage factors are D_1_ and D_2_, respectively [26], and the material completely failed when the damage factor was 1. The degradation relationship could be linear, exponential, parabolic, and have other functional relationships, but different degradation methods had little impact on the structure. Considering the balance of accuracy and calculation cost, the linear degradation met the requirements.

### 2.2. Materials and Models

**Figure 2:**
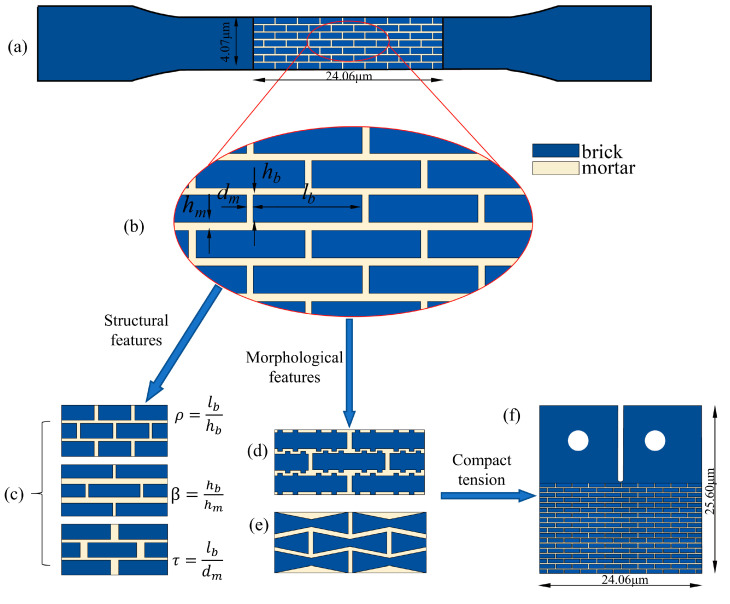
Schematic diagram of nacre model: (**a**) tensile test model, (**b**)regular structure, (**c**) three structural parameters, (**d**) racked morphology, (**e**) wedged morphology, (**f**) c-t model.

A 2D numerical model of a standard tensile specimen of size 24.06 × 4.7 μm was established, as shown in Figure 2. In the model with regular unit, the mortar thickness *h_m_* was much less than brick thickness *h_b_*, brick length *l_b_* was much larger than the brick thickness as described in reference, *d_m_* was the distance between two adjacent bricks at the same level [13]. Three characteristic parameters were defined as variables, namely aspect ratio $\rho =\frac{l_{b}}{h_{b}}$, thickness ratio $\beta =\frac{h_{b}}{h_{m}}$, and spacing ratio $\tau =\frac{l_{b}}{d_{m}}$, as shown in Figure 2c.

Each brick in the racked structure (Figure 2d) had four uniformly distributed bumps with a bump amplitude of 0.1 μm; the thickness of the brick in the wedged structure (Figure 2e) decreased linearly from 0.4 μm to 0.2 μm, the brick length in the wedged and racked structures was 8 μm and the mortar thickness was 0.03 μm. In addition, three models of compact tension specimen for cyclic loading were established with a size of 24.06 × 25.6 μm in Section 3.4 as shown in Figure 2f. 

The modeling and calculation of the research contents of this paper were performed in ABAQUS. The model was divided into two sets by partitioning the part, and the two sets were assigned different material properties. To equivalent the complex interface failure behavior of B-M structure and make the macroscopic mechanical performance of the model conform to the actual situation, the material parameters of mortar have been revised. The assignment of material properties were exhibited in Table 1. The coefficient of viscous stability for the maximum principal stress damage was 1e^−5^, with a tolerance of 0.005. Larger viscosity coefficients and tolerances could improve the convergence of the models, but the accuracy of the results will be reduced. The parameters used in the models can successfully complete the calculation with guaranteed accuracy. To improve the computational efficiency, four-node plane strain quadrilateral elements (CPE4) were used in the finite element model. The entire B-M model was set up as an enriched region to adapt to the real expansion trajectory of the fissures. Considering the effect of crack location and depth on the results, there was no pre-fabricated cracking of the model. The displacement control method was used as the loading mode, which effectively reduced the sharp change in stress during the crack expansion process and thus improved model convergence. To assure mesh quality, partitioned meshing was used for distinct models with the same mesh size. It is worth mentioning that the XFEM level set functions PHILSM and PSILSM of the models need to be output in the analysis step.

## 3. Results and Discussions

### 3.1. Model Validation

**Figure 3:**
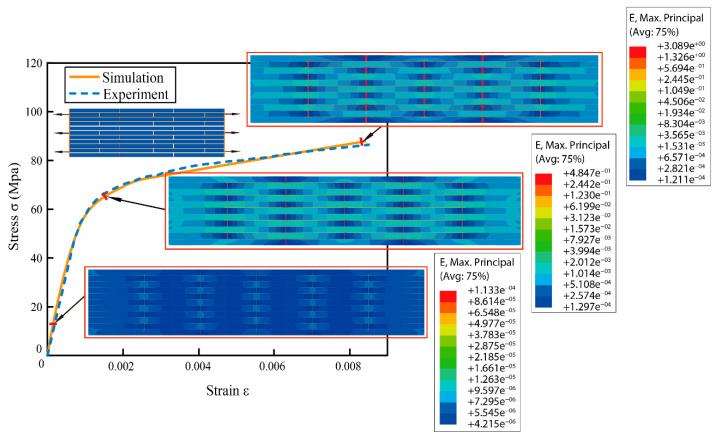
Comparison of the experimental results in the references and the simulation results of the current finite element model. The inset figures depicted snapshots of the strain in the model at 10 MPa, 65 MPa, and 85 MPa stresses, respectively.

To verify our structural model and calculation method were available and accurate, the mechanical performance of a bioinspired nacreous layer was simulated and compared with the experimental results which were carried out by Barthelat et al. [13] as shown in Figure 3. A regular staggered structure was established to study the tensile behavior in our structural model, of which structural parameters and materials properties were obtained from the published work [27,28]. The simulation results showed the maximum failure stress was 3% larger than that of the experimental test, and the failure strain was 1.17% smaller than the experimental result. The total strain including elastic strain and plastic strain were exhibited along the stress-strain curve. At the beginning stage, only elastic deformation emerged, and plastic deformation occurred when strain reached nearly 0.0016 and stress was over 60 MPa. With the continuous increase of total strain, the stress increased gradually and reached the yield limit as the result of plastic deformation accumulated. Finally, our structural model fractured when the ultimate stress reached about 85 MPa and failure strain reached 0.0085. Thus, the results obtained from our numerical model fitted well with the experimental results, and had a good calculation accuracy which could be used to study the effect of different structural parameters on strength and toughness.

### 3.2. Structural Effect on Strength and Toughness

**Figure 4:**
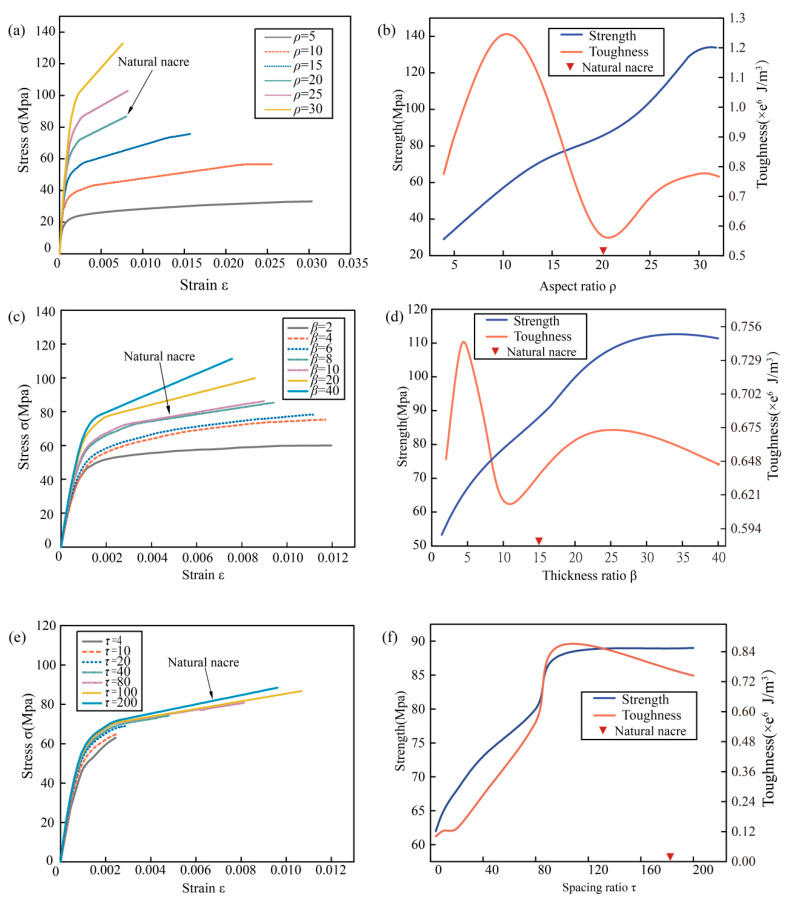
Stress-strain and strength-toughness curves with different structural parameters: (**a**) tensile behavior with different ρ, (**b**) effect of ρ on strength and toughness, (**c**) tensile behavior with different β, (**d**) effect of β on strength and toughness, (**e**) tensile behavior with different τ, (**f**) effect of τ on strength and toughness.

It is well-known that the staggered B-M microstructure contributed more than the properties of materials did to the final mechanical performance of the nacreous layer. Thus, three characteristic structural parameters, including ρ, β, and τ, were used to study the connection between microstructure and mechanical performance. The tensile behavior of numerical models with different structural parameters were exhibited in Figure 4a,c,e, and both the strength and toughness were calculated and were presented in Figure 4b,d,f. Toughness was defined as the energy absorbed by a material before fracture, and could be evaluated analytically by integrating the area under the stress-strain curve [29]. 

The toughness calculating formulation was described as

(5) $$T=\int_{0}^{\varepsilon f}\sigma d\varepsilon ,$$

where *T* was the energy absorbed per unit volume, *σ* was the stress, *ε* was the strain, and *εf* was the failure strain. 

Figure 4a showed stress-strain curves of B-M structure with different ρ. It could be found directly that the fracture strength increased from 30 MPa to 130 MPa with an increase of ρ from 5 to 30. On the contrary, the fracture strain decreased from 0.03 to 0.008 which proved deformation capacity decreased as ρ increasing, but the decreasing tendency became stable when ρ was larger than 20. The effect of aspect ratio ρ on strength and toughness was completely different. The tensile strength increased approximately linearly within the variation range of ρ, but the toughness increased initially to the peak 1.25 MJ/m^3^, then it decreased to the valley 0.55 MJ/m^3^. The tendency of toughness accompanying with ρ was similar to a sinusoidal function as shown in Figure 4b. Thus, it was difficult to improve strength and toughness at the same time by optimizing aspect ratio ρ. The effect of thickness ratio β on strength and toughness was close to that of aspect ratio ρ as shown in Figure 4d. With the growth of β, the increasing tendency of strength was approximately linear, and the varying tendency of toughness was also close to a sinusoidal function. The maximum toughness was 0.74 MJ/m^3^ when β was 5, while the minimum toughness was 0.6 MJ/m^3^ when β was 10. The fracture strength increased from 53 MPa to 110 MPa with the growing of β, and the fracture strain decreased from 0.012 to 0.008. Although the effect tendency of ρ and β on strength and toughness was similar, the effect degree of ρ was larger than that of β. However, the effect of spacing ratio τ on strength and toughness was completely different from that of ρ and β. Both strength and toughness increased almost simultaneously as τ increased, the fracture strength increased from 62 MPa to 89 MPa, and the toughness increased from 0.12 MJ/m^3^ to 0.84 MJ/m^3^ with a little retracement at the end of the curve. Thus, it could be concluded that the effect of ρ on strength and toughness was more significant than the other two structural parameters, while, by increasing τ, the strength and toughness could be improved simultaneously.

### 3.3. Morphology Effect on Strength and Toughness

**Figure 5:**
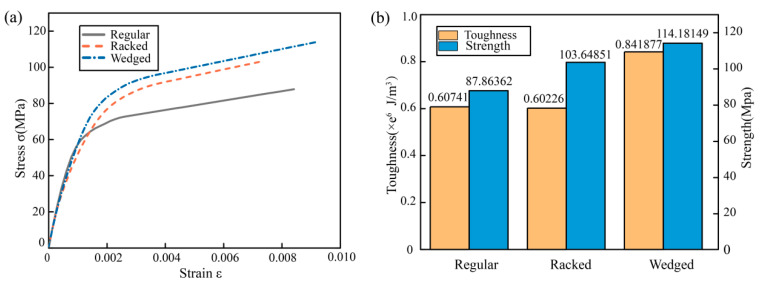
Tensile simulation results of different morphological models: (**a**) stress-strain curves, (**b**) Strength and toughness.

The actual microstructure of the nacreous layer was more sophisticated than the uniform staggered B-M structure. Many researchers revealed its true morphology, and found a great variety of microstructures existed within 19 species, such as columnar nacre, sheet nacre, foliated, prismatic, cross-lamellar, and complex cross-lamellar [30,31]. To further study the relationship between the unit morphology and mechanical performance, three types of staggered unit morphology were extracted from the former studies, called the regular unit, racked unit, and wedged unit. When building numerical models with these three types of morphology, the aragonite volume fraction was kept the same in each model. Tensile simulations of the model with different morphology units were carried out, and the stress-strain curves were exhibited in Figure 5a. It was clear that the model with the wedged unit possessed the strongest fracture strength and the largest tensile strain. Moreover, the fracture strength of the model with racked unit was 17.97% higher than that with regular unit, the tensile strain was smaller due to the analogical action of gear meshing. Toughness of these three models was also calculated and are shown in Figure 5b, it could be found that the toughness of the model with wedged unit was also the largest at about 0.842 MJ/m^3^, and the toughness of the other two models was very close to each other at about 0.6 MJ/m^3^. Thus, the staggered unit with wedged morphology could improve the toughness by 38.61% and the strength by 29.95%, which was the most significant in affecting the strength and toughness. 

**Figure 6:**
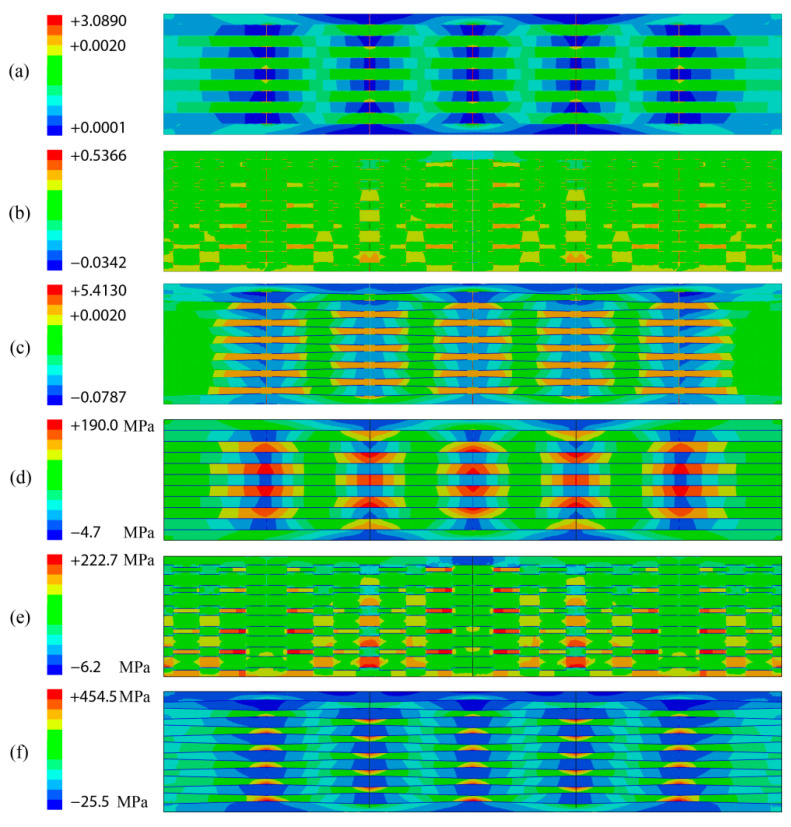
Horizontal stress and strain of different morphological models: (**a**) horizontal strain of the regular model, (**b**) horizontal strain of the racked model, (**c**) horizontal strain of the wedged model, (**d**) horizontal stress of the regular model; (**e**) horizontal stress of the racked model, (**f**) horizontal stress of the wedged model.

To further explain the tensile behavior of these three models, the distribution of horizontal strain and stress were, respectively, presented in Figure 6. The strain distributions of the regular model and the wedged model were similar to each other, and the largest deformation occurred in organic components between the adjacent mineral bricks due to the lower elastic modulus of organic components. Additionally, the strain distribution of the racked model was different from the other two models, because the racked morphology could resist large deformation in horizontal direction as the similar function of gear meshing. It also showed that the strain of racked model was the smallest which was in good accordance with the result of tensile simulation as shown in Figure 5a. This was also similar to the report that the nano and micro bulge in reference [10] could provide shear resistance at a very small sliding distance. This indicated that other microstructure features were the main source of the bond strength between the staggered unit over a large sliding distance of the nacre. The largest strain was generated in the model with wedged unit morphology, because the strain area increased by the inclined surface. This wedged inclined surface endured considerable inelastic deformation and had a certain strain hardening effect, making the pearl layer more ductile, and the sheet bevel was an important source of shear resistance [32,33]. Moreover, the stress distributions found in these three models were also different as shown in Figure 6d–f. It was easy to find that the stress distribution in the regular model and racked model was more uniform, there was no obvious stress concentration that happened in the local region compared with the stress distribution of the wedged model. Thus, the stress concentration found in the wedged model was the largest due to the strain being the largest among these three models. The wedged morphology was more likely to cause greater deformation than the other two models.

### 3.4. Morphology Effect on Crack Resistance

**Figure 7:**
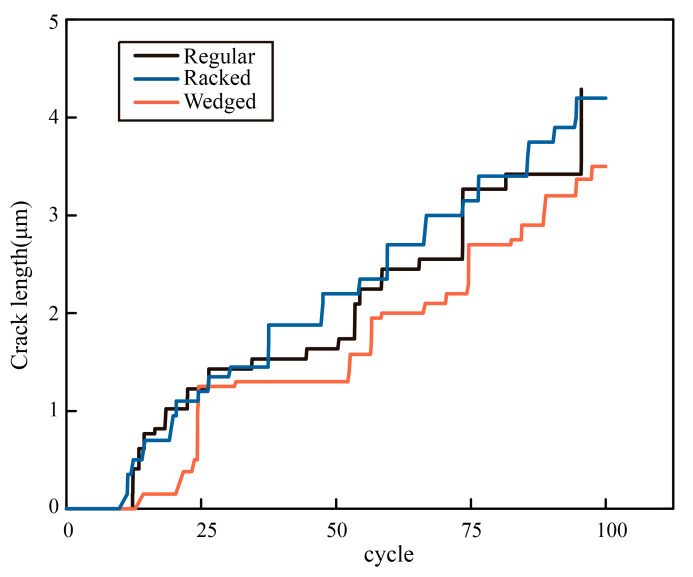
Crack growth curves corresponding to different morphological models.

The compact tension models were used to study crack propagation in different microstructures in order to find out which unit morphology was the best in resisting crack growing, as the ability to resist crack growing was an important indicator for evaluating toughness. The crack depth as the function of loading cycles was recorded in Figure 7. It was found that the crack depth of the model with the wedged unit was the shortest after the same loading cycles. The crack depths of the other two models were longer, and the growing tendency was similar. Moreover, the crack depth of the wedged model suspended growing during 25 cycles to 50 cycles, which also proved its ability to resist crack growing. 

**Figure 8:**
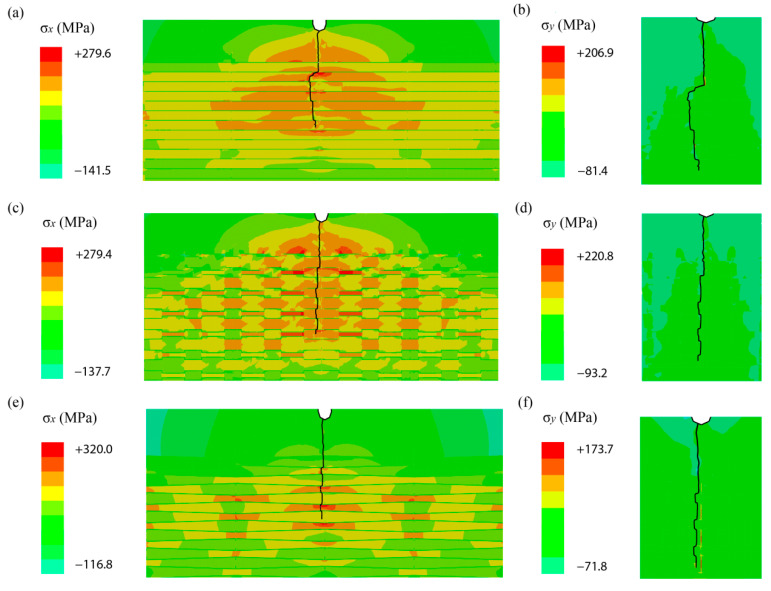
Crack growing path and stress distribution of different morphological models: (**a**) horizontal stress of the regular model, (**b**) vertical stress of the regular model, (**c**) horizontal stress of the racked model, (**d**) vertical stress of the racked model, (**e**) horizontal stress of the wedged model, (**f**) vertical stress of the wedged model.

To illustrate the resistance of crack path, the distribution of horizontal stress and strain which led to the growing of crack path was presented in Figure 8. Observing the stress distribution in the model with regular unit, it was found that the crack deflection was more obvious than that in the other two models, due to the area of horizontal stress was larger and more stress concentrated around the crack. To be more specific, the crack grew directly in aragonite at the initial stage, and changed the growing direction the first time it reached the interface between the aragonite and organic layers. A similar conclusion was reached by other researchers which was that an oscillating or mismatch of elastic modulus could prolong the crack path. However, the crack gradually returned to its original direction along the symmetrical axis of the model. While observing the stress distribution in the models with racked unit and wedged unit, the areas of stress concentration were smaller and were distributed more dispersed by the unit morphology. It was found that the maximum stress occurred on both sides of crack in the racked model, and the maximum stress was found in front of the crack in the wedged model. The maximum stress generated in these three models were 279.6 MPa, 279.4 MPa, and 320 MPa, respectively, in aragonite, which were very close to the results obtained by Barthelat [13]. These relatively high stresses emphasized the importance of unit morphology in B-M structure. The maximum stress could also be transmitted into the organic layer, which could enhance the energy absorption as the result of increasing plasticizing strain. In addition, the wedged unit resembled a dovetail, a locking device widely used in mechanical assemblies to prevent tablet sliding and pullout by generating additional resistance through compression of the sheet. Among these three models, the horizontal strain of the model with wedged tablets was the smallest, and that of the regular model was the largest, which was in good accordance with the results of crack depth. Thus, the wedged tablet was proved to be the best in resisting crack growth for the B-M model. 

**Figure 9:**
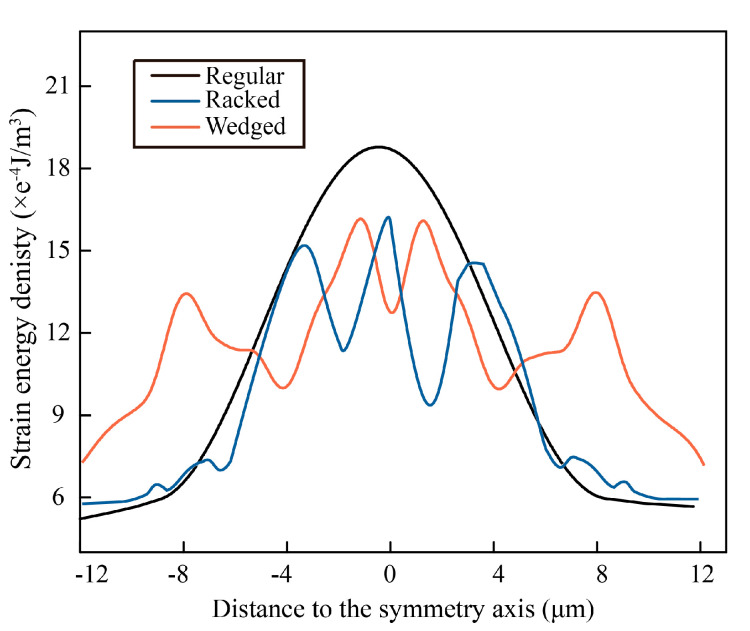
Strain energy density in various morphological models.

To explain the crack path, the strain energy density perpendicular to the crack was studied and it is presented in Figure 9. It could be found easily that the strain energy density peak of regular model was the largest about 19e^−4^ J/m^3^, and the peak was also the widest, which indicated the largest strain generated around the crack path. Compared with the distribution of strain energy density found in the other two models, it could be speculated the total strain was smaller, and the destroyed energy was absorbed more uniformly by the wedged model.

**Figure 10:**
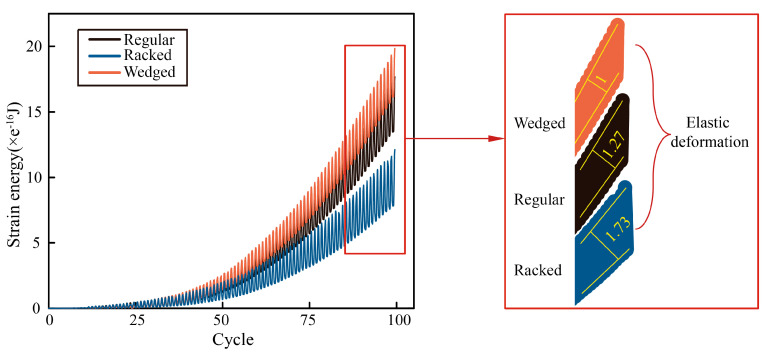
Strain energy of different morphological models.

In addition, Figure 10 showed the strain energy absorbed by the three models during loading cycles. It indicated that the strain energy absorbed by the wedged model was the largest, and the strain energy absorbed by the regular model was more than in the racked model. This result could also be concluded by the strain distribution of these three models (Figure 6a–c), as the strain generated in the wedged model was the largest during single tensile process. Moreover, the amplitude of single cycle that occurred in the wedged model was the smallest which indicated elastic deformation was smaller than that of the other two models. It could be concluded that plastic strain energy absorbed by the wedged model was the largest, and that absorbed by the racked model was the smallest, because, the total strain in the racked model was also the smallest, as shown in Figure 6b.

## 4. Conclusions

The extended finite element method was used to investigate the connection between mechanical behavior and bionic structure inspired by the nacre. The practicality and accuracy of the established numerical model were examined by comparing the simulation results with the reported experimental results. Three characteristic structural parameters, such as aspect ratio, thickness ratio, and spacing ratio were, respectively, used as variables to investigate the effect of different microscopic structures on the strength and toughness. The results showed the aspect ratio was the most prominent factor in determining the strength and toughness of the nacre. Although the influence law of thickness ratio on strength and toughness was similar to that of the aspect ratio, the influence degree was relatively smaller. The effect of spacing ratio was quite different, which could improve the strength and toughness of bioinspired nacreous structure at the same time. Moreover, three types of morphologies of the nacreous unit, such as the regular unit, racked unit, and wedged unit, were established to reveal their effect on the mechanical performance. It was found that the strength and toughness of the wedged model were improved the most compared with that of the regular and racked models. The compact tensile models with these three morphologies unit were established to further study the effect of the nacreous unit morphology on fractured crack resistance. By analyzing the fractured crack path and fracture energy, it was found that the wedged morphology of the nacreous unit was the best in resisting crack propagation. 

Finally, this paper presented the connection between the structure and mechanical performance of bionic structures inspired by the nacre, which was significant for designing high performance composite materials and engineering structures. Meanwhile, the simulation method used in this paper provided an available technique to study mechanical behavior of bioinspired structures.

## Figures and Tables

**Table 1:** Mechanical properties of nacre in finite element model [27,28].

Material	Young’s Modulus (GPa)	Poisson’s Ratio	Critical Fracture Energy (MPa·m)	Strength (MPa)
Brick	100	0.25	0.104	140
Mortar	4	0.23	0.026	40

## Data Availability

Data will be made available upon reasonable request.
